# Prodromal symptoms and the duration of untreated psychosis in first episode of psychosis patients: what differences are there between early vs. adult onset and between schizophrenia vs. bipolar disorder?

**DOI:** 10.1007/s00787-023-02196-7

**Published:** 2023-04-07

**Authors:** Inmaculada Baeza, Elena de la Serna, Gisela Mezquida, Manuel J. Cuesta, Eduard Vieta, Silvia Amoretti, Antonio Lobo, Ana González-Pinto, Covadonga M. Díaz-Caneja, Iluminada Corripio, Isabel Valli, Olga Puig, Anna Mané, Miquel Bioque, Miriam Ayora, Miquel Bernardo, Josefina Castro-Fornieles, the PEPs group, Clemente García-Rizo, Jairo González-Díaz, Mario de Matteis, Héctor de Diego, Eva Grasa, Alejandra Roldán, Iñaki Zorrilla, Edurne García-Corres, Pedro M Ruíz-Lázaro, Concepción de-la-Cámara, Olga Rivero, María José Escarti, Francesc Casanovas, Alba Toll, Norma Verdolini, Maria Sagué-Vilabella, Gisela Sugranyes, Daniel Ilzarbe, Fernando Contreras, Leticia González-Blanco, María Paz García-Portilla, Miguel Gutierrez, Arantzazu Zabala, Roberto Rodríguez-Jiménez, Luis Sánchez-Pastor, Judith Usall, Anna Butjosa, Edith Pomarol, Salvador Sarró, Angela Ibáñez, Ana Maria Sánchez-Torres, Vicent Balanzá-Martínez

**Affiliations:** 1 Child and Adolescent Psychiatry and Psychology Department, Hospital Clínic de Barcelona, 2021SGR01319 Barcelona, Spain; 2 Centro de Investigación Biomédica en Red de Salud Mental, CIBERSAM-ISCIII, Madrid, Spain; 3 Institut d’Investigacions Biomèdiques August Pi Sunyer (CERCA-IDIBAPS), Barcelona, Spain; 4 Department of Medicine, Universitat de Barcelona, Barcelona, Spain; 5 Barcelona Clinic Schizophrenia Unit, Hospital Clinic of Barcelona, Neuroscience Institute, Hospital Clínic de Barcelona, Barcelona, Spain; 6 Department of Psychiatry, Complejo Hospitalario de Navarra, Pamplona. IdiSNA, Navarra Institute for Health Research, Pamplona, Spain; 7 Bipolar and Depressive Disorder Unit, Neuroscience Institute, Hospital Clínic de Barcelona, Barcelona, Spain; 8 Group of Psychiatry, Psychiatric Genetics Unit, Mental Health and Addictions, Vall d’Hebron Research Institute (VHIR), Barcelona, Spain; 9 Department of Medicine and Psychiatry, Hospital Clínico Universitario and Instituto de Investigación Sanitaria (IIS) Aragón, Universidad de Zaragoza, Zaragoza, Spain; 10 Department of Psychiatry, Hospital Universitario de Alava, BIOARABA, EHU, Vitoria, Spain; 11 Department of Child and Adolescent Psychiatry, Institute of Psychiatry and Mental Health, School of Medicine, Hospital General Universitario Gregorio Marañón, Universidad Complutense, IiSGM, Madrid, Spain; 12 Psychiatry Department, Institut d’Investigació Biomèdica-Sant Pau (IIB-SANT PAU), Hospital de La Santa Creu I Sant Pau, Universitat Autònoma de Barcelona (UAB), Barcelona, Spain; 13 Department of Psychosis Studies, Institute of Psychiatry, Psychology and Neuroscience, King’s College London, London, UK; 14 Hospital del Mar Medical Research Institute (IMIM), Pompeu Fabra University, Barcelona, Spain

**Keywords:** Schizophrenia, bipolar disorder, early onset, Adult onset, Children and adolescents, Prodrome, Prodromal symptoms

## Abstract

To assess the role of age (early onset psychosis-EOP < 18 years vs. adult onset psychosis-AOP) and diagnosis (schizophrenia spectrum disorders-SSD vs. bipolar disorders-BD) on the duration of untreated psychosis (DUP) and prodromal symptoms in a sample of patients with a first episode of psychosis. 331 patients with a first episode of psychosis (7–35 years old) were recruited and 174 (52.6%) diagnosed with SSD or BD at one-year follow-up through a multicenter longitudinal study. The Symptom Onset in Schizophrenia (SOS) inventory, the Positive and Negative Syndrome Scale and the structured clinical interviews for DSM-IV diagnoses were administered. Generalized linear models compared the main effects and group interaction. 273 AOP (25.2 ± 5.1 years; 66.5% male) and 58 EOP patients (15.5 ± 1.8 years; 70.7% male) were included. EOP patients had significantly more prodromal symptoms with a higher frequency of trouble with thinking, avolition and hallucinations than AOP patients, and significantly different median DUP (91 [33–177] vs. 58 [21–140] days; Z = − 2.006, p = 0.045). This was also significantly longer in SSD vs. BD patients (90 [31–155] vs. 30 [7–66] days; Z = − 2.916, p = 0.004) who, moreover had different profiles of prodromal symptoms. When assessing the interaction between age at onset (EOP/AOP) and type of diagnosis (SSD/BD), avolition was significantly higher (Wald statistic = 3.945; p = 0.047), in AOP patients with SSD compared to AOP BD patients (p = 0.004). Awareness of differences in length of DUP and prodromal symptoms in EOP vs. AOP and SSD vs. BD patients could help improve the early detection of psychosis among minors.

**Supplementary Information:**

The online version contains supplementary material available at 10.1007/s00787-023-02196-7.

## Introduction

In recent years, a considerable effort has been made to diagnose and treat the early stages of psychosis, focusing on patients with At-Risk Mental States (ARMS) and first-episode of psychosis (FEP) [1–4]. This strategy aims to detect prodromal symptoms of psychosis as early as possible in order to decrease the time between the onset of psychosis, the diagnosis and the initiation of treatment (duration of untreated psychosis, DUP) [5]. The DUP has been found to be neurotoxic [6, 7] and longer DUP has been associated with a worse outcome in patients with FEP in most studies (for review [8, 9]). In schizophrenia, the longer the DUP, the poorer the outcome (clinical, social and global) [10]. Moreover, it is well known that the early manifestation of schizophrenia in childhood and adolescence has a poorer prognosis than adult onset (for a review [11]), and a longer DUP is also a predictor of worse outcome in this population [12], although this has not been described in adult patients with Bipolar Disorder (BD) [13].

Most DUP studies have not distinguished between early onset psychosis (EOP), where the illness is diagnosed before 18 years of age [14], and adult onset psychosis (AOP). When looking at age, some studies did report longer DUP in EOP vs. AOP [15–17], while others found the opposite [18, 19], but younger age did not specifically mean EOP in all of the studies [15–19].

DUP has been reported to be shorter in some studies including FEP patients with affective disorders [20]; for a review, [21], but not in other original studies [22, 23]. Specifically, shorter DUP has been described as a predictor of bipolar disorder (BD) vs. schizophrenia in FEP patients [24–26]. However, there is a lack of information about at the role of early age at onset when comparing the DUP of Schizophrenia Spectrum Disorders (SSD) and BD.

Prodromal symptoms of psychosis could be different between children and adolescents vs. adults due to neurodevelopmental characteristics [27]. In the general population, children and adolescents aged 8–15 have more unusual perceptual experiences and attenuated hallucinations than older subjects [28], and child and adolescent ARMS mostly describe perceptual abnormalities and suspiciousness [29]. No study to date has systematically compared prodromal symptoms in EOP vs. AOP samples. However, the need to adapt the ARMS approach to study the specific traits of these mental states in childhood and adolescence has been highlighted [30].

Moreover, prodromal symptoms could be different between SSD and BD patients [31], and investigating these possible differences could help clinicians respond to the needs of FEP patients with greater precision. A few studies have reviewed schizophrenia prodrome as well as bipolar prodrome [32–34], but comparison studies are scarce. In adults, no differences have been observed in prodromal symptoms between ARMS patients who develop SSD or affective psychosis [35]. In adolescents, some prodromal symptoms such as suspiciousness were more frequent in subjects who went on to develop schizophrenia, while impulsivity, suicidal thoughts, sleeplessness and extreme energy were described in youngsters who subsequently developed bipolar disorder[36].

To the best of our knowledge, no previous studies have compared the DUP and characteristics of prodromal symptoms between EOP vs. AOP and between SSD and BD, while taking the age at onset into account.

### Aims of the study

To assess the role of age and diagnosis (SSD vs. BD) on the duration of untreated psychosis and prodromal symptoms in a sample of patients with a first episode of psychosis.

## Material and methods

16 centers in Spain participated in a 2-year prospective longitudinal naturalistic multicenter study conducted between 2009 and 2012 in which 335 patients with a FEP and 253 matched healthy controls were included (for a full description of the study design see [37, 38]). Most of the centers were part of the well-recognized Spanish network of research in Mental Health named “Centro de Investigación Biomédica en Red de Salud Mental (CIBERSAM)” [39].

### Subjects

From the whole sample (N = 335), only FEP patients who had Symptom Onset in Schizophrenia (SOS) inventory data were included (N = 331). To assess the DUP and type of prodromal symptoms in SSD and BD patients as well as the interaction between diagnosis and age at onset in these patients, the diagnosis made for each patient at the one-year follow-up assessment was accepted as definitive (N = 174). The other patients who were not included in this analysis either continued to have a diagnosis of FEP (N = 74) or had missed (N = 83) the one-year assessment.

Each patient who met the inclusion criteria at any of the participating sites was invited to take part in the study. Inclusion criteria were: (1) age between 7 and 35 years, (2) presence of psychotic symptoms which had begun within the previous 12 months, (3) fluency in Spanish and (4) signed informed consent. Exclusion criteria were: (1) intellectual disability according to DSM-IV criteria [40], (2) history of head trauma with loss of consciousness and (3) presence of an organic disease with mental repercussions.

The study was approved by the ethics review committee of each participating center, following the principles of the Declaration of Helsinki. Informed consent was obtained from all participants as well as their parents or legal guardians if they were underage.

### Assessment

The assessments were performed by experienced psychiatrists or psychologists and the following data were obtained:

-Sociodemographic data, including the Socioeconomic Status (SES), measured with the Hollingshead and Redlich scale [41].

- Family psychiatric background, registered through an interview with the parents or legal guardians.
- Symptom Onset of Schizophrenia (SOS) inventory [42], validated Spanish version [43]. It includes 16 items grouped into 4 subscales: general prodromal (7 items), negative (4), positive (2) and disorganized (2) symptoms. There is also an “Other” category for symptoms that are not included in the other subscales, such as magical thinking or hoarding. Each item is scored from 0 (never) to 4 (continuously present), according to the severity and persistence of the symptom, and is rated at the highest frequency that the symptom occurs. If any symptom reaches a certain frequency threshold criterion, which is different for each symptom (e.g. 4 for sleeping problems, ≥ 1 for perceptual abnormalities, etc.) then the date when the symptom crossed the frequency threshold is registered.Date of onset of psychosis is recorded according to the subject, the treating clinician and a family member. In our study, DUP was calculated as the number of days between the first manifestations of psychotic symptoms indicated by the clinician until the initiation of adequate treatment for psychosis.
- Diagnoses were made using the Spanish version of the Structured Clinical Interview for DSM-IV Axis I disorders (SCID-I) [44] for AOP or the Spanish version of the Schedule for Affective Disorders and Schizophrenia for School-Aged Children (K-SADS) [45, 46] for EOP.
- Positive and Negative Syndrome Scale (PANSS), validated Spanish version [47, 48], a 30 item scale measuring positive, negative and general symptoms of schizophrenia, which are scored from 1 (not present) to 7 (severe).
- Cannabis use was assessed using the adapted version of the Multidimensional Assessment Instrument for Drug and Alcohol Dependence scale [49].

### Procedures

At baseline, the SOS inventory was administered to the patient, one family member or legal guardian if available, and the treating clinician. The assessment was completed with the rest of the clinical scales mentioned above. One-year follow-up assessment diagnoses were classified into 2 categories: SSD (schizophreniform disorder; schizophrenia; schizoaffective disorder and delusional disorder) and BD (bipolar disorder I, manic or depressive episode with psychotic features).

### Data analysis

To describe the sample, we used continuous variables expressed as means, standard deviations (SD) and ranges, and categorical variables expressed as frequencies and/or percentages. DUP was compared between the groups using the median because it was not normally distributed, and non-parametric tests (Mann–Whitney U and Kruskal Wallis H tests) were used in the analysis. Median was described including the percentiles: [25th percentile, 75th percentile]. Sociodemographic data were compared using the Student t test for continuous variables and χ^2^ test for categorical ones. Generalized linear models were used to compare clinical variables between the groups (EOP vs.. AOP and SSD vs. BD) as main effects for all comparisons. Moreover, we used generalized linear models to assess the interaction between the groups. Statistics were performed with IBM® SPSS 25.0 for Windows. Differences of p < 0.05 were considered significant. We did not correct for multiple comparisons, and because of this we consider our findings exploratory.

## Results

### Sociodemographic and clinical characteristics in the EOP vs. AOP sample

Among the 331 patients included in the study, 273 were AOP and 58 EOP. Table 1 shows the sociodemographic and clinical assessment of both subsamples. The number of adoptees (χ^2^ = 9.437, p = 0.030) and personal psychiatric background (χ^2^ = 27.798, p < 0.001) were statistically higher in the EOP vs. AOP sample, but no other significant differences were found between the groups.

**Table 1:** Sociodemographic and clinical characteristics of the sample according to patients’ age at the onset of psychosis

	EOP patients N = 58	AOP patients N = 273	t/χ^2^/Z/Wald statistic	p
Age (years) (mean ± SD)	15.5 ± 1.8	25.2 ± 5.1	− 14.250	** < 0.001**
Sex (N,% male)	41 (70.7)	181 (66.5)	0.373	0.664
SES (mean ± SD)	2.9** ± **1.5	3.1** ± **1.3	− 1.098	0.273
Adoptee (N,%)	2 (3.4)	0	9.437	**0.030**
Personal psychiatric background (N,%)	37 (63.8)	78 (28.6)	27.798	** < 0.001**
Current Cannabis abuse or dependence (N,%)	11 (19)	56 (20.5)	0.859	0.475
Familial psychotic history (1st degree) (N,%)	7 (12.1)	25 (9.2)	0.464	0.319
DUP (days) [25th percentile, 75th percentile]	91 [33–177]	58 [21–140]	− 2.006	**0.045**
SOS general, number of symptoms (mean ± SD)	4.1 ± 1.4	3.5 ± 1.6	8.012	**0.005**
SOS negative, number of symptoms (mean ± SD)	1.2 ± 1.3	0.8 ± 1.1	6.060	**0.014**
SOS positive, number of symptoms (mean ± SD)	1.8 ± 0.4	1.5 ± 0.6	7.539	**0.006**
SOS disorganized, number of symptoms (mean ± SD)	0.9 ± 0.9	0.9 ± 0.9	0.006	0.937
SOS total, number of symptoms (mean ± SD)	8 ± 3	6.7 ± 2.8	9.134	**0.003**
PANSS-P scores (mean ± SD)	19.6 ± 8.2	18.5 ± 7.9	1.094	0.296
PANSS-N scores (mean ± SD)	18.9 ± 9.9	18.5 ± 7.7	0.385	0.701
PANSS-G scores (mean ± SD)	36.8 ± 16.4	37.6 ± 11.8	0.195	0.659
PANSS-T scores (mean ± SD)	78.6 ± 29.5	74.3 ± 23.4	1.446	0.229

*AOP* adult onset psychosis, *EOP* early onset psychosis, *PANSS* positive And negative syndrome scale, *P* positive, *N* negative, *G* general, *T* total, *SD* standard deviation, *SES* socioeconomic status, *SOS* symptom onset of schizophrenia

### Prevalence of type of prodromal symptoms and DUP in EOP and AOP patients

Median DUP showed significant differences in EOP vs. AOP patients (91 [33–177] vs. 58 [21–140] days; Z = − 2.006, p = 0.045) (Table 1). When the sample was stratified into groups of ≤ 13 years (very early onset; N = 4, 180.5 [135–464] days); ≥ 13–17 years (N = 54, 76 [30–170] days) and AOP (58 [21–140] days), there was also a trend toward significance in median DUP (χ^2^ = 5.203, p = 0.074). No differences were observed when comparing median DUP between sexes in EOP (male/female: 90 [45–172] and 91 [24–260] days) and AOP patients (male/female: 61 [21–150] and. 47 [24–137] days) (χ^2^ = 4.579; p = 0.205).

Globally, no differences were detected in median DUP between patients with and without a first-degree family history of psychosis in both EOP (with/without: 107 [57–204] and 120 [35–176] days) and AOP patients (with/without: 61 [30–136] and. 60 [14–148] days) (χ^2^ = 4.170; p = 0.244).

**Fig. 1:**
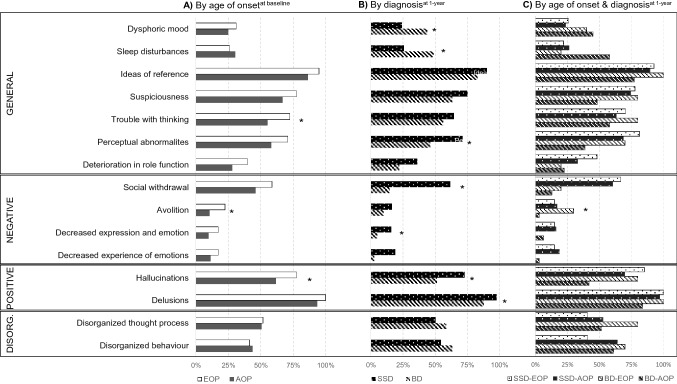
Percentage of prodromal symptoms measured with the Symptom Onset in Schizophrenia (SOS) inventory according to the (**A**) the age of onset of psychosis, (**B**) type of diagnosis at one-year assessment, and (**C**) the age at onset and type of diagnosis at one-year assessment. Footnote. *EOP* early onset psychosis, *AOP* adult onset psychosis, *SSD* schizophrenia Spectrum Disorder; *BD* = Bipolar disorder, *DISORG* disorganized. (**A**) N(EOP) = 58 and N(AOP) = 27; (**B**) N(SSD) = 133 and N(BD) = 41; C) N(SSD-EOP) = 27, N(SSD-AOP = 106), N(BD-EOP) = 10 and N(BD-AOP) = 31; *p < .05

Prodromal symptoms from the SOS inventory are described in Table 1, Fig. 1 and Supplementary Table 1. EOP patients experienced more prodromal symptoms ( 8 ± 3.1) than AOP (6.7 ± 2.8, Wald = 9.134, p = 0.003). Moreover, there were differences in the frequency of prodromal symptoms between the subsamples for the following symptoms: trouble with thinking (Wald = 5.813, p = 0.016), avolition (Wald = 6.189, p = 0.013) and hallucinations (Wald = 5.196, p = 0.023), which were more prevalent in EOP than AOP subjects.

### Baseline sociodemographic and clinical characteristics, prevalence of DUP and type of prodromal symptoms in SSD and BD patients

From the 331 patients at baseline, 248 (74.9%) had a one-year assessment, and 174 of them (70.2%) were diagnosed with some SSD (N = 133) or BD (N = 41) and included in our analysis. No differences were found in sociodemographic characteristics, PANSS subscales or in total scores at baseline between patients who were assessed at one-year follow-up and those who were not (Supplementary Table 2).

Sociodemographic characteristics of SSD and BD patients at one year assessment are shown in Table 2, without significant differences between the groups. Median DUP was longer in SSD vs. BD (90 [31–155] vs. 30 [7–66] days; Z = − 2.916, p = 0.004) (Table 2). Dysphoric mood (Wald = 5.833, p = 0.016) and sleep disturbance (Wald = 7.586, p = 0.006) were significantly more frequent in BD than SSD patients, while perceptual abnormalities (Wald = 8.373, p = 0.004), hallucinations (Wald = 6.544, p = 0.011), delusions (Wald = 5.664, p = 0.017), social withdrawal (Wald = 22.070, p < 0.001) and decreased experience of emotions (Wald = 4.400, p = 0.036) were more prevalent in SSD than BD patients (Fig. 1 and Supplementary Table 3).

**Table 2:** Baseline sociodemographic and clinical characteristics of the sample according to patients’ diagnosis at one-year of follow-up

	SSD patients N = 133	BD patients N = 41	t/χ^2^/Z/Wald statistic	p
Age (years) (mean ± SD)	23.9 ± 5.9	23 ± 6.2	0.862	0.390
Sex (N,% male)	43 (32.3)	11 (26.8)	0.443	0.567
SES (mean ± SD)	3.1 ± 1.3	3.1** ± **1.3	0.184	0.854
Adoptee (N,%)	1 (0.8)	1 (3.4)	0.785	0.417
Personal psychiatric background (N,%)	44 (33.1)	19 (46.3)	3.884	0.056
Current Cannabis abuse or dependence (N,%)	24 (18)	7 (17.1)	0.020	1.000
Familial psychotic history (1st degree) (N,%)	15 (11.3)	4 (9.8)	0.075	1.000
DUP (days) [25th percentile, 75th percentile]	90 [31–155]	30 [7–66]	2.916	**0.004**
SOS general, number of symptoms (mean ± SD)	3.9 ± 1.5	3.6 ± 1.2	0.923	0.357
SOS negative, number of symptoms (mean ± SD)	1.1 ± 1.3	0.3 ± 0.6	3.967	** < 0.001**
SOS positive, number of symptoms (mean ± SD)	1.7 ± 0.5	1.4 ± 0.7	3.378	**0.001**
SOS disorganized, number of symptoms (mean ± SD)	1 ± 0.9	1.1 ± 0.9	0.679	0.498
SOS total, number of symptoms (mean ± SD)	7.7 ± 2.9	6.4 ± 2.2	2.559	**0.011**
PANSS-P scores (mean ± SD)	19.4 ± 7.3	19.7 ± 8.7	0.202	0.840
PANSS-N scores (mean ± SD)	21.4 ± 8	14.5 ± 6.9	6.967	** < 0.001**
PANSS-G scores (mean ± SD)	39.7 ± 12.8	36.5 ± 14.2	1.329	0.186
PANSS-T scores (mean ± SD)	81.5 ± 23.6	70.7 ± 24.8	2.527	**0.012**

*BD* bipolar disorder, *PANSS* positive and negative syndrome scale, *P* positive, *N* negative, *G* general, *T* total, *SD* standard deviation, SES socioeconomic status, *SOS* symptom onset of schizophrenia, *SSD* schizophrenia spectrum disorders

### Interaction of the age at onset by type of diagnosis on DUP and type of prodromal symptoms

**Fig. 2:**
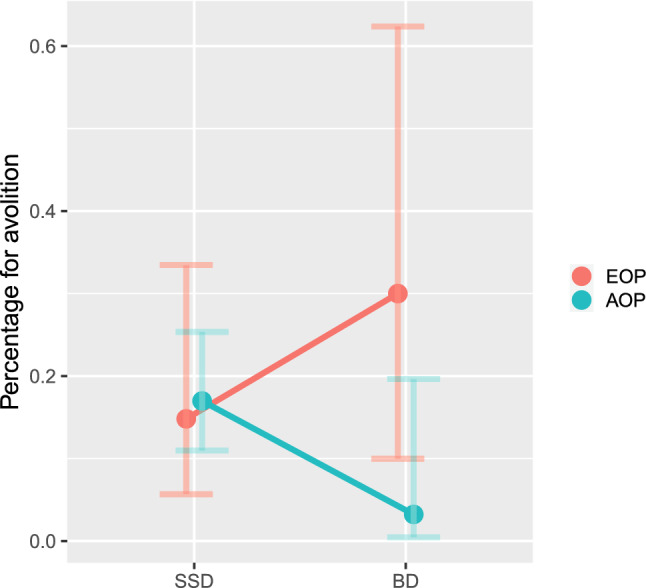
Probability of the prodromal symptom avolition according to age at onset and type of diagnostic at one-year of follow-up

Among the 174 patients assessed at one year, 37 were EOP (27 SSD and 10 BD) and 137 were AOP (106 SSD and 31 BD). When interaction of the age at onset (EOP/AOP) by type of diagnosis (SSD/BD) were assessed, the symptom avolition was statistically significant (Wald = 3.945, p = 0.047), with AOP patients with SSD having a higher frequency of this symptom compared to AOP BD patients (p = 0.004) (Fig. 2 and Supplementary Table 4).

## Discussion

The goal of this study was to elucidate differences in the type of prodromal symptoms and DUP among FEP patients when age at onset of psychosis (EOP/AOP) and diagnostic outcome at 1- year follow-up (SSD/BD) were taken into account. EOP had significantly more prodromal symptoms and a higher frequency of symptoms such as trouble with thinking, avolition and hallucinations. EOP patients also showed a significant longer median DUP compared to AOP patients. At the same time, DUP was significantly longer in SSD vs. BD patients, with each group having a different profile regarding the frequency of specific prodromal symptoms compared to SSD. When interaction of the age at onset (EOP/AOP) by type of diagnosis (SSD/BD) was assessed, the symptom avolition was significantly higher in AOP with SSD vs. AOP BD patients.

The model of early intervention in psychosis is focused on the detection of prodromal symptoms in order to reduce the DUP [4]. Longer periods of untreated psychosis have been found to affect the short and long-term outcome of FEP patients (for review [50]). However research in this area has been limited by factors such as the lack of a psychometrically standardized definition of DUP and the use by some clinicians of retrospective clinical measurement of DUP with no scale or systematic method [5]. Our study tried to address these limitations using the SOS Inventory [42], an instrument which has been reported to be a reliable way to measure the onset of psychosis [51].

In our study, EOP patients showed a significant longer median DUP compared to AOP. This is consistent with Dominguez et al. [52], who also found longer DUP in adolescents vs. adult FEP (179 vs. 86 days) and other previous studies which have described longer mean DUP in EOP vs. AOP such as: 2.6 ± 4.1 vs.1 ± 2.5 years[14]; 77 ± 135 vs. 33.2 ± 67.5 weeks [53]; and 103.6 ± 162.3 vs. 46.3 ± 70.1 weeks[54]. Moreover, in the one study where even younger patients (≤ 13 years) were taken into account [14], this population had longer DUP than EOP and AOP, which is similar to our findings. These studies used heterogeneous methods of measuring DUP: the Circumstances of Onset and Relapse Schedule [55] in Ballageer et al.[54]; the shortened version of the Nottingham Onset Schedule [56] in Dominguez et al. [52] and no clear structured instrument in Coulon et al. [14] and Joa et al. [53], but the methods of measuring the onset and end-point of DUP did not contribute to the heterogeneity of the mean or median DUP values in a systematic review [21]. Nevertheless, the findings of these previous studies are consistent with our own in that all seem to suggest that identifying prodromal psychotic symptoms in children and adolescents is more difficult than in adults, and this may be the reason for the difference in DUP.

Years ago, McGlashan [57] stated that DUP appeared to be the product of different forces such as denial of illness by the patient and family, paranoid views regarding mental health treatment, negative symptoms with loss of motivation which impede individuals from seeking treatment, as well as insidiously unfolding psychosis. Nowadays, it is possible that these reasons persist more in children and adolescents than in adults. To increase the effectiveness of early intervention programs, efforts should be made to change these attitudes and encourage families and young individuals to seek help as soon as possible.

Among other factors, some genetic variations have previously been found to have no association with DUP [58]. This is consistent with our data which shows no differences in median DUP between EOP and AOP patients with or without a first-degree family history of psychosis. Nevertheless, in our study, no genetic analyses were performed; instead only the reported family history of psychosis was taken into account. In adults, longer DUP has been found in patients with a greater family history of psychosis [59], although other authors found only longer duration of untreated illness, but not of DUP, in patients with a first episode of psychotic disorder and a family history of psychosis vs. those without this family history [60]. In either case, these findings seem to indicate that a previous family experience of psychosis might not contribute the recognition of the need for help. Considering the importance of rapid detection and treatment, this is an important issue which warrants further study.

A significantly higher number of prodromal symptoms measured with the SOS inventory was found in EOP compared to AOP patients, with higher frequency of trouble with thinking, avolition and hallucinations in EOP subjects. No other studies with samples of EOP and AOP FEP have compared these measures. In young adults with SSD, a mean of 7.5 prodromal symptoms based on the Instrument for the Retrospective Assessment of Onset of Schizophrenia were identified, with impaired role functioning and social withdrawal being the most prevalent [61]. This study also found a much lower prevalence of prodromal unusual perceptual experiences (28.1%) than in our sample, where the prevalence was 68.9% [61]. However, some symptoms of ARMS, such as perceptual abnormalities and suspiciousness, seem to have different prevalence rates in younger patients [29]. In the general population, subjects with ARMS criteria between the ages of 8 and 40 years, showed an age effect on the occurrence of attenuated positive symptoms, and perceptual abnormalities in particular [28]. Before the age of around 16, individuals were more likely to report attenuated perceptual abnormalities such as unusual perceptual experiences and hallucinations [28].

Some differences in the clinical presentation of prodromal symptoms of FEP could be framed in the developmental model which describes the ethiopathology of psychosis [62, 63] and could explain the higher frequency of hallucinations in younger subjects. Other factors such as obstetric complications, premorbid intelligence quotient < 85 and personal psychiatric background [64] or cortical thickness [65], may also be more relevant prodromal symptoms in younger patients. The current study shares patients with a separate, previously published study [64] which also found that EOP subjects were more likely to have a personal psychiatric background or to be adoptees than the AOP sample. This supports the notion that there could be a higher genetic load in younger subjects [66, 67]. Taking this into account could facilitate the early detection of psychosis.

Looking at the one-year diagnosis of BD vs. SSD, in our study patients with BD had significantly shorter DUP than those who developed SSD. This is similar to what has been reported by other authors [20; 24–26], and might be associated with the type of prodromal and psychotic symptoms that lead to earlier consultation of mental health professionals. Looking at the duration of the prodromal stage, Kafali et al. [36] reported no differences between adolescents with SSD and BD. When examining the prevalence of prodromal symptoms, these authors found a greater prevalence of suspiciousness in adolescent patients with SSD than in those with BD. This contrasts somewhat with our findings which showed increased perceptual abnormalities, hallucinations, delusions, social withdrawal and decreased experience of emotions in SSD compared to BD patients. These findings are consistent with what has been reported in adult patients, where social isolation or withdrawal, marked impairment of role functioning and personal hygiene and marked lack of initiative, interests, or energy were more prevalent prodromal symptoms in schizophrenia than in BD patients [68]. Similar symptoms were also described in SSD adult patients, with the most prevalent being marked isolation, impairment of role functioning, preoccupation and marked lack of initiative, interests or energy [69].

Prodromal symptoms for schizophrenia had been part of some DSM criteria prior to DSM-IV, but they were omitted due to their lack of specificity compared to other psychotic disorders [40, 68]. At the same time, studies have also found a higher prevalence of certain prodromal symptoms in patients with BD. Our study found that dysphoric mood and sleep disturbance were significantly more prevalent in BD than in SSD patients. Both symptoms are consistent with the mania prodrome (for a review [70, 71]), although not all bipolar patients included in the review had psychotic symptoms. Focusing on adolescents, Kafali et al. [36] found that apart from sleeplessness, other attenuated manic symptoms such as extreme energy and inflated self-esteem or behavioral disturbances (oppositionality, temper tantrums) were more prevalent in the prodromal stage of BD compared to SSD patients. Correll et al. [72] also identified similar symptoms in the prodrome of young BD patients, although the comparison group in this article was based on previous studies of SSD prodrome by other authors. In adolescents with an ARMS, those who developed BD reported more perceptual abnormalities as prodromal symptoms, while those with SSD described more disorganized communication, although the authors stated that bipolar prodrome might be indistinguishable from the schizophrenia prodrome [73].

Despite this caveat, the findings suggest that prodromal symptoms differ between subjects who later develop SSD vs. BD. However, the relevant studies all relate to subjects whose ages were similar. Our study aimed to determine what additional trends might be found by grouping subjects according to their age at onset in addition to their diagnostic outcome. When we assessed interaction of the age at onset (EOP/AOP) by type of diagnoses (SSD/BD), only prodromal avolition was statistically significant, with AOP with SSD having a higher frequency of this symptom compared to AOP BD patients. Avolition is a reduction in the initiation of and persistence in goal-directed activities and the desire to perform such activities, and it is considered a negative symptom [74]. It has been reported to be the strongest and most reliable predictor of certain elements of functional outcome [75]. Network analytic findings indicate that it is a highly central symptom which is interconnected with other negative symptom domains in schizophrenia [75]. It may be difficult to distinguish avolition from other negative or depressive symptoms [76], however doing so could help clinicians identify adult patients who are likely to later develop a SSD. Negative prodromal symptoms in the early stages of psychosis are known to be predictors of short term outcome in first-episodes of psychosis [77]. Our findings offer additional evidence that it could be helpful for early detection and prevention programs to focus closely on these symptoms.

In summary, the results from the current study suggest that taking patients’ age into account when assessing prodromal symptoms may help clinicians intervene as precisely as possible, reduce the DUP and improve the response to treatment [78]. The focus on different prodromal symptoms in children and adolescents vs. adults could help early intervention programs more effectively respond to their patients’ needs.

### Limitations

This study has several limitations. First, the sample was reduced after being divided into the categories of EOP vs. AOP patients as well as between BD and SSD patients and there were differences in the sample sizes of the groups which could have affected the results. Also, only 9 of the 16 (56.3%) participant centers were able to recruit child and adolescent patients with an EOP. Similarly, we could not include a very early onset psychosis (onset < 13 years) subsample due to the small number of patients in this age range (N = 4). Patients were interviewed in an unstructured way to complete the inventory, and the Structured Clinical Interview for the scale (SCI-SOS) was not used. Another limitation is that while the SOS inventory provides a great deal of detailed information regarding the prodromal period of psychosis, it does not determine the date when the patient first met criteria for a psychotic disorder. Moreover, we used the SOS inventory to assess all patients with a first episode of psychosis, and this included patients with BD who are not the intended target of this instrument. Lastly, we did not correct for multiple comparisons in the analysis, and, as such, we consider our study exploratory.

### Strengths

First, this study has a considerably large and homogeneous FEP sample size, which includes both adolescent and adult participants. Moreover, the sample was prospectively recruited, which helps to generalize the results. An additional strength is that we used a validated scale of prodromal symptoms to assess both the symptoms and the DUP, to help overcome one of the limitations of previous studies in this field.

### Supplementary Information

Below is the link to the electronic supplementary material.

Supplementary file1 (DOCX 18 KB)

Supplementary file2 (DOCX 18 KB)

Supplementary file3 (DOCX 18 KB)

Supplementary file4 (DOCX 20 KB)

## Data Availability

The data that support the findings of this study are available from the corresponding author, IB, upon reasonable request.
