# Supplementary material for: Prodromal symptoms and the duration of untreated psychosis in first episode of psychosis patients: what differences are there between early vs. adult onset and between schizophrenia vs. bipolar disorder?
Source: Eur Child Adolesc Psychiatry. 2023 Apr 7;33(3):799–810. doi: 10.1007/s00787-023-02196-7 (PMC10894175; doi:10.1007/s00787-023-02196-7)
Supplement: Supplementary file 1 — Supplementary file1 (DOCX 18 KB) [file 787_2023_2196_MOESM1_ESM.docx]

Supplementary Table 1. Description of the prodromal symptoms measured using the Symptom Onset in Schizophrenia (SOS) inventory according to the age of onset of psychosis.

|  | **EOP**  **patients**  **N=58** | **AOP**  **patients**  **N= 273** | **Wald Statistic** | **p** |
| --- | --- | --- | --- | --- |
| **GENERAL PRODROMAL SYMPTOMS*** | | | | |
| **Dysphoric mood (N,%)** | 18 (31) | 68 (24.9) | 0.929 | 0.335 |
| **Sleep disturbances (N,%)** | 15 (25.9) | 82 (30) | 0.401 | 0.526 |
| **Ideas of reference (N,%)** | 55 (94.8) | 235 (86.1) | 3.091 | 0.079 |
| **Suspiciousness (N, %)** | 45 (77.6) | 182 (66.7) | 2.803 | 0.107 |
| **Trouble with thinking (N,%)** | 42 (72.4) | 150 (54.9) | 5.813 | **0.016** |
| **Perceptual abnormalities (N,%)** | 41 (70.7) | 158 (57.9) | 3.223 | 0.073 |
| **Deterioration in role function (N,%)** | 23 (39.7) | 76 (27.8) | 3.142 | 0.076 |
| **NEGATIVE PRODROMAL SYMPTOMS** | | | | |
| **Social withdrawal (N,%)** | 34 (58.6) | 125 (45.8) | 3.116 | 0.078 |
| **Avolition (N,%)** | 13 (22.4) | 28 (10.3) | 6.189 | **0.013** |
| **Decreased expression of emotion (N,%)** | 10 (17.2) | 26 (9.5) | 2.853 | 0.091 |
| **Decreased experience of emotions (N,%)** | 10 (17.2) | 30 (11) | 1.730 | 0.188 |
| **POSITIVE PRODROMAL SYMPTOMS** | | | | |
| **Hallucinations (N,%)** | 45 (77.6) | 168 (61.5) | 5.196 | **0.023** |
| **Delusions (N,%)** | 58 (100) | 255 (93.4) | 0.001 | 0.998 |
| **DISORGANIZED PRODROMAL SYMPTOMS** | | | | |
| **Disorganized thought process (N,%)** | 30 (51.7) | 138 (50.5) | 0.028 | 0.871 |
| **Disorganized behavior (N,%)** | 24 (41.4) | 119 (43.6) | 0.095 | 0.758 |

AOP: Adult Onset Psychosis; EOP: Early Onset Psychosis. *The item “Other” of the inventory has too big heterogeneity and has been not shown in the table.
