# Supplementary material for: Prodromal symptoms and the duration of untreated psychosis in first episode of psychosis patients: what differences are there between early vs. adult onset and between schizophrenia vs. bipolar disorder?
Source: Eur Child Adolesc Psychiatry. 2023 Apr 7;33(3):799–810. doi: 10.1007/s00787-023-02196-7 (PMC10894175; doi:10.1007/s00787-023-02196-7)
Supplement: Supplementary file 2 — Supplementary file2 (DOCX 18 KB) [file 787_2023_2196_MOESM2_ESM.docx]

Supplementary Table 2. Sociodemographic and clinical characteristics of the sample comparing subjects who underwent the one-year follow-up assessment and those who did not.

|  | **Patients**  **with 1-year follow-up**  **assessment**  **N=248** | **Patients**  **without 1-year follow-up**  **assessment**  **N=83** | **t/χ^2^Wald statistic** | **p** |
| --- | --- | --- | --- | --- |
| **Age (years)**  **(mean±SD)** | 23.7±5.8 | 25.2±6.3 | -1.934 | 0.054 |
| **Sex (N,% male)** | 174 (70.1) | 49 (59) | 2.308 | 0.136 |
| **SES (mean±SD)** | 3.1**±**1.3 | 3.2**±**1.4 | -0.747 | 0.456 |
| **Adoptee (N,%)** | 2 (0.8) | 0 | 3.736 | 0.154 |
| **Personal psychiatric background (N,%)** | 83 (33.5) | 28 (33.7) | 0.495 | 0.485 |
| **Familial psychotic background (1^st^ degree) (N,%)** | 24 (9.7) | 8 (9.6) | 0.005 | 1.000 |
| **PANSS-P scores (mean±SD)** | 18.5 ±7.9 | 19.2±8.2 | -0.762 | 0.447 |
| **PANSS-N scores (mean±SD)** | 18.9±8.1 | 17.7±8.1 | 1.142 | 0.254 |
| **PANSS-G scores (mean±SD)** | 37.5±13 | 37.2±11.9 | 0.199 | 0.842 |
| **PANSS-T scores (mean±SD)** | 75.4±24.8 | 73.9±24.1 | 0.481 | 0.631 |

AOP: Adult Onset Psychosis; EOP: Early Onset Psychosis; PANSS: Positive And Negative Syndrome Scale; P: positive; N: negative; G: general; T: total; SD: Standard Deviation; SES: Socioeconomic Status.
