# Supplementary material for: Prodromal symptoms and the duration of untreated psychosis in first episode of psychosis patients: what differences are there between early vs. adult onset and between schizophrenia vs. bipolar disorder?
Source: Eur Child Adolesc Psychiatry. 2023 Apr 7;33(3):799–810. doi: 10.1007/s00787-023-02196-7 (PMC10894175; doi:10.1007/s00787-023-02196-7)
Supplement: Supplementary file 3 — Supplementary file3 (DOCX 18 KB) [file 787_2023_2196_MOESM3_ESM.docx]

Supplementary Table 3. Description of the prodromal symptoms measured using the Symptom Onset in Schizophrenia (SOS) inventory according to the type of diagnosis at one-year assessment.

|  | **SSD**  **patients**  **N=133** | **BD**  **patients**  **N= 41** | **Wald statistic** | **p** |
| --- | --- | --- | --- | --- |
| **GENERAL PRODROMAL SYMPTOMS*** | | | | |
| **Dysphoric mood (N,%)** | 32 (24.1) | 18 (43.9) | 5.833 | **0.016** |
| **Sleep disturbances (N,%)** | 34 (25.6) | 20 (48.8) | 7.586 | **0.006** |
| **Ideas of reference (N,%)** | 120 (90.2) | 34 (82.9) | 1.601 | 0.206 |
| **Suspiciousness (N, %)** | 100 (75.2) | 26 (63.4) | 2.146 | 0.143 |
| **Trouble with thinking (N,%)** | 86 (64.7) | 23 (56.1) | 0.977 | 0.323 |
| **Perceptual abnormalities (N,%)** | 95(71.4) | 19 (46.3) | 8.373 | **0.004** |
| **Deterioration in role function (N,%)** | 48 (36.1) | 9 (22) | 2.777 | 0.096 |
| **NEGATIVE PRODROMAL SYMPTOMS** | | | | |
| **Social withdrawal (N,%)** | 82 (61.7) | 6 (14.6) | 22.070 | **<0.001** |
| **Avolition (N,%)** | 22 (16.5) | 4 (9.8) | 1.108 | 0.292 |
| **Decreased expression of emotion (N,%)** | 21 (1915.8) | 2 (4.9) | 2.887 | 0.089 |
| **Decreased experience of emotions (N,%)** | 24 (19) | 1 (2.4) | 4.400 | **0.036** |
| **POSITIVE PRODROMAL SYMPTOMS** | | | | |
| **Hallucinations (N,%)** | 97 (72.9) | 21 (51.2) | 6.544 | **0.011** |
| **Delusions (N,%)** | 130 (97.7) | 36 (87.8) | 5.664 | **0.017** |
| **DISORGANIZED PRODROMAL SYMPTOMS** | | | | |
| **Disorganized thought process (N,%)** | 67 (50.4) | 24 (58.5) | 0.833 | 0.361 |
| **Disorganized behavior (N,%)** | 79 (54.4) | 26 (63.4) | 0.107 | 0.744 |

BD: Bipolar Disorder; SSD: Schizophrenia Spectrum Disorders. * The item “Other” of the inventory showed too much heterogeneity to be included in this table.
