# Supplementary material for: Prodromal symptoms and the duration of untreated psychosis in first episode of psychosis patients: what differences are there between early vs. adult onset and between schizophrenia vs. bipolar disorder?
Source: Eur Child Adolesc Psychiatry. 2023 Apr 7;33(3):799–810. doi: 10.1007/s00787-023-02196-7 (PMC10894175; doi:10.1007/s00787-023-02196-7)
Supplement: Supplementary file 4 — Supplementary file4 (DOCX 20 KB) [file 787_2023_2196_MOESM4_ESM.docx]

Supplementary Table 4. Comparison of the Duration of Untreated Psychosis (DUP) and prodromal symptoms,

measured using the Symptoms Onset in Schizophrenia (SOS) inventory, according to the age at onset and type

of diagnosis at one-year assessment.

|  | **Schizophrenia Spectrum Disorder Patients**  **N=133** | | **Bipolar Disorder**  **patients**  **N=41** | | **Wald Statistic** | **p for interaction** |
| --- | --- | --- | --- | --- | --- | --- |
|  | **EOP**  **N=27** | **AOP**  **N=106** | **EOP**  **N=10** | **AOP**  **N=31** |  |  |
| **DUP (median, 25^th^ percentile, 75^th^ percentile, days)** | 127 [34-183] | 91 [39-183] | 43 [4-205] | 30 [6-80] | 0.004 | 0.951 |
| **GENERAL PRODROMAL SYMPTOMS** | | | | | | |
| **Dysphoric mood (N,%)** | 7 (25.9) | 25 (23.6) | 4 (40) | 14 (45.2) | 0.143 | 0.705 |
| **Sleep disturbances (N,%)** | 6 (22.2) | 28 (26.4) | 2 (20) | 18 (58.1) | 2.157 | 0.142 |
| **Ideas of reference (N,%)** | 25 (92.6) | 95(89.6) | 10 (100) | 24 (77.4) | 0.001 | 0.999 |
| **Suspiciousness (N, %)** | 21 (77.8) | 79 (74.5) | 8 (80) | 18 (58.1) | 0.761 | 0.383 |
| **Trouble with thinking (N,%)** | 19 (70.4) | 67 (63.2) | 8 (80) | 15 (48.4) | 1.306 | 0.253 |
| **Perceptual abnormalities (N,%)** | 22 (81.5) | 73 (68.9) | 7 (70) | 12 (38.7) | 0.425 | 0.514 |
| **Deterioration in role function (N,%)**** | 13 (48.1) | 35 (33) | 2 (20) | 7 (22.6) | 0.620 | 0.431 |
| **NEGATIVE PRODROMAL SYMPTOMS** | | | | | | |
| **Social withdrawal (N,%)** | 18 (66.7) | 64 (60.4) | 2 (20) | 4 (12.9) | 0.058 | 0.812 |
| **Avolition (N,%)** | 4 (14.8) | 18 (17) | 3 (30) | 1 (3.2) | 3.945 | **0.047** |
| **Decreased expression of emotion (N,%)** | 4 (14.8) | 17 (16) | 0 | 2 (6.5) | 0.001 | 0.999 |
| **Decreased experience of emotion (N,%)** | 4 (14.8) | 20 (18.9) | 0 | 1 (3.2) | 0.001 | 0.999 |
| **POSITIVE PRODROMAL SYMPTOMS** | | | | | | |
| **Hallucinations (N,%)** | 23 (85.2) | 74 (69.8) | 8 (80) | 13 (41.9) | 0.585 | 0.444 |
| **Delusions (N,%)** | 27 (100) | 103 (97.2) | 10 (100) | 26 (83.9) | 0 | 1.000 |
| **DISORGANIZED PRODROMAL SYMPTOMS** | | | | | | |
| **Disorganized thought process (N,%)** | 11 (40.7) | 56 (52.8) | 8 (80) | 16 (51.6) | 3.463 | 0.063 |
| **Disorganized behavior (N,%)** | 11 (40.7) | 68 (64.2) | 7 (70) | 19 (61.3) | 2.095 | 0.148 |

AOP: Adult Onset Psychosis; EOP: Early Onset Psychosis; DUP: Duration of Untreated Psychosis.
